# Patterns and Effects of Admission Hyperglycemia and Inflammatory Response in Trauma Patients: A Prospective Clinical Study

**DOI:** 10.1007/s00268-021-06190-5

**Published:** 2021-06-11

**Authors:** Ayman El-Menyar, Mohammad Asim, Fayaz Mir, Suhail Hakim, Ahad Kanbar, Tariq Siddiqui, Basil Younis, Khalid Ahmed, Ismail Mahmood, Sajid Atique, Hisham Al Jogol, Ibrahim Taha, Fuad Mustafa, Mohammad Alabdallat, Husham Abdelrahman, Ruben Peralta, Hassan Al-Thani

**Affiliations:** 1 Clinical Medicine, Weill Cornell Medical College, Doha, Qatar; 2 Trauma & Vascular Surgery, Clinical Research, Hamad General Hospital, Doha, Qatar; 3 Qatar Metabolic Institute, Hamad Medical Corporation, Doha, Qatar; 4 Department of Surgery, Trauma Surgery, Hamad General Hospital, Doha, Qatar; 5 Department of Surgery, Universidad Nacional Pedro Henriquez Urena, Santo Domingo, Dominican Republic

## Abstract

**Background:**

The constellation of the initial hyperglycemia, proinflammatory cytokines and severity of injury among trauma patients is understudied. We aimed to evaluate the patterns and effects of on-admission hyperglycemia and inflammatory response in a level 1 trauma center. We hypothesized that higher initial readings of blood glucose and cytokines are associated with severe injuries and worse in-hospital outcomes in trauma patients.

**Methods:**

A prospective, observational study was conducted for adult trauma patients who were admitted and tested for on-admission blood glucose, hemoglobin A1c, interleukin (IL)-6, IL-18 and hs-CRP. Patients were categorized into four groups [non-diabetic normoglycemic, diabetic normoglycemic, diabetic hyperglycemic (DH) and stress-induced hyperglycemic (SIH)]. The inflammatory markers were measured on three time points (admission, 24 h and 48 h). Generalized estimating equations (GEE) were used to account for the correlation for the inflammatory markers. Pearson’s correlation test and logistic regression analysis were also performed.

**Results:**

During the study period, 250 adult trauma patients were enrolled. Almost 13% of patients presented with hyperglycemia (50% had SIH and 50% had DH). Patients with SIH were younger, had significantly higher Injury Severity Score (ISS), higher IL-6 readings, prolonged hospital length of stay and higher mortality. The SIH group had lower Revised Trauma Score (*p* = 0.005), lower Trauma Injury Severity Score (*p* = 0.01) and lower GCS (*p* = 0.001). Patients with hyperglycemia had higher in-hospital mortality than the normoglycemia group (12.5% vs 3.7%; *p* = 0.02). A significant correlation was identified between the initial blood glucose level and serum lactate, IL-6, ISS and hospital length of stay. Overall rate of change in slope 88.54 (95% CI:-143.39–33.68) points was found more in hyperglycemia than normoglycemia group (*p* = 0.002) for IL-6 values, whereas there was no statistical significant change in slopes of age, gender and their interaction. The initial IL-6 levels correlated with ISS (*r* = 0.40, *p* = 0.001). On-admission hyperglycemia had an adjusted odds ratio 2.42 (95% CI: 1.076–5.447, *p* = 0.03) for severe injury (ISS > 12) after adjusting for age, shock index and blood transfusion.

**Conclusions:**

In trauma patients, on-admission hyperglycemia correlates well with the initial serum IL-6 level and is associated with more severe injuries. Therefore, it could be a simple marker of injury severity and useful tool for patient triage and risk assessment.

**Trial registration:**

This study was registered at the ClinicalTrials.gov (Identifier: NCT02999386), retrospectively Registered on December 21, 2016. https://clinicaltrials.gov/ct2/show/NCT02999386.

**Supplementary Information:**

The online version contains supplementary material available at 10.1007/s00268-021-06190-5.

## Introduction

Hyperglycemia following trauma is a hypermetabolic response to stress which can be associated with a significant morbidity and mortality [1]. Some investigators have suggested that admission glucose level could be used as a potential predictor of hospital outcomes as it reflects the physiological stress reaction to injury, bleeding and shock [2–4]. However, there is still a need to have a consensus on the definition and measurement of stress-induced hyperglycemia (SIH) in trauma patients [2].

The trauma-related metabolic surge and associated SIH were found to correlate with serum cortisol and catecholamine levels [5]. It has been suggested that insulin production is suppressed in trauma patients due to systemic stress response secondary to elevated serum glucagon, catecholamine and cytokines [6–8].

Assessment of glycosylated hemoglobin (HbA1c) level is considered as a useful tool to distinguish occult (not known before) diabetes mellitus (DM) from SIH [9]. Kopelman et al. [10] reported that 18% of trauma patients initially presented with hyperglycemia, of which 22% had occult DM which represented 4% of the total screened trauma patients. The possible mechanism of the adverse effects of hyperglycemia may differ in patients with SIH as compared to diabetic hyperglycemia (DH). SIH is an acute process, initiated by the release of stress hormones and cytokines, while DH is a chronic condition associated with subsequent microvascular changes [11].

Moreover, there is a relationship between hyperglycemia and altered cellular metabolism in critically ill patients that results in insulin resistance and release of systemic inflammatory mediators [6]. Earlier studies have suggested that proinflammatory cytokines such as interleukin (IL)-6 and IL-18 are involved in glucose metabolism and insulin action; therefore, hypercytokinemia may have a potential role in increased glucose levels [6, 12–14]. As most observations on the association of cytokines with hyperglycemia are based on experimental studies, there is a need to explore such relationship with respect to the clinical presentation and outcome in trauma patients. Herein, this prospective study aims to evaluate the patterns and effects of on-admission hyperglycemia, proinflammatory cytokines and severity of injury in trauma patients. We hypothesized that higher initial readings of blood glucose and cytokines are associated with severe injuries and worse in-hospital outcomes in trauma patients.

## Materials and methods

A prospective observational study was conducted for trauma patients who were admitted to the level 1 trauma center at Hamad General Hospital (HGH) between October 2016 and July 2019. Inclusion criteria were adult (≥ 18 years) trauma patients (all genders) presented to the emergency department and were investigated for random blood glucose level and HbA1C within 5 h of hospital admission. Exclusion criteria included patients declined to participate or in whom random glucose level and HbA1C were not measured on time, vulnerable populations (children, pregnant women) and alcohol consumers. All trauma patients underwent thorough clinical assessment and resuscitation according to the Advanced Trauma Life Support (ATLS) guidelines. Potential subjects were enrolled after obtaining written informed consent either by subject or his/her next-of-kin or deferred consent for blood investigations and use of data with secured confidentiality of personal information.

### Study variables

Data included patients’ demographics, (age, gender, nationality), mechanism of injury, initial vitals (heart rate, respiratory rate, systolic blood pressure, diastolic blood pressure and shock index), routine laboratory findings such as hemoglobin, base deficit, serum lactate and blood glucose levels were recorded at the baseline, after 24 h and 48 h. Other investigations included white blood cell count (WBC), platelet count, high-sensitive troponin T (hs-TnT), HbA1C and blood ethanol levels. We have collected information about history of DM, anti-diabetic medications, associated injuries, Injury Severity Score (ISS), Glasgow Coma Score (GCS), Revised Trauma Score (RTS), Trauma Injury Severity Score (TRISS), surgical intervention, blood transfusion, hospital length of stay, intensive care unit stay, in-hospital complications (pneumonia, acute respiratory distress syndrome, renal failure and sepsis) and hospital mortality. Shock index (SI) was defined as initial heart rate divided by the initial systolic blood pressure.

The main exposure was hyperglycemia, defined as random serum glucose 200 mg/dL (11.1 mmol/l) or more. This cutoff level of glucose was previously used to define hyperglycemia by earlier studies in trauma patients [15–17]. DM was determined by patient history and/or admission HbA1c ≥ 6.5% (≥ 48 mmol/mol). This level of HbA1c is based on current recommendations for the diagnosis of DM from the American Diabetes Association [18]. SIH was defined as hyperglycemia on admission in patients with normal HbA1c in the index admission [19].

### Serum levels of CRP, IL-6 and IL-18

Enzyme-linked immunosorbent assay (ELISA) was performed using commercially available kits for cytokine detection (R&D) Systems. The preparation of all reagents, the working standards and protocol were followed according to the manufacturer’s instructions. The absorbance was read using ELISA reader (TECAN) at 450 nm and 570 nm using dual filters. The minimum detectable dose was 0.005 ng/ml for hs-CRP, 0.7 pg/ml for IL-6 and 1.25 pg/ml for IL-18. All the samples were thawed only once and assayed in duplicate.

### Statistical analysis

Sample size was calculated considering the prevalence of SIH in trauma patients that ranges from 10 to 17% for all trauma admissions [15, 20] with a precision of estimate (margin of error) of 5% and a 95% level of confidence. Using the single proportion equation for dichotomous variables in the nMaster 2.0 sample size software package, the required sample size was 250 consecutive trauma patients.

**Fig. 1:**
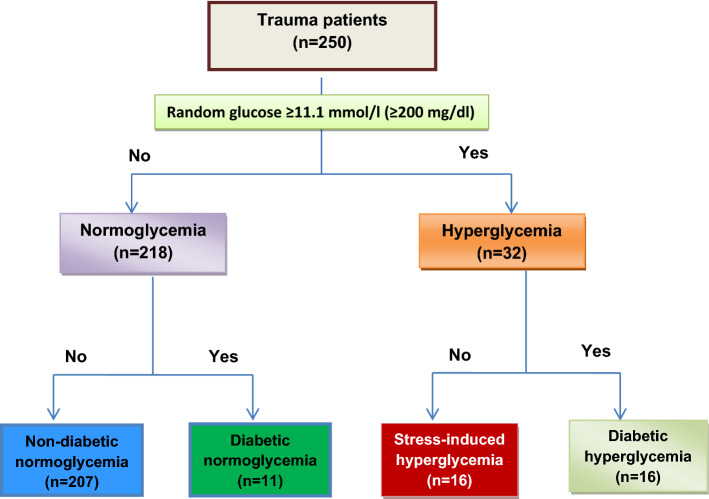
Study design

Data were reported as proportion, mean (± standard deviation), confidence intervals, median and interquartile range (IQR), whenever applicable. Assessment of normality of continuous data was performed using Shapiro–Wilk test. The mean values of IL-6, IL-18, hs-CRP and blood glucose at baseline after 24 h and 48 h were expressed as mean and 95% confidence interval (95% CI). The levels of blood glucose, serum lactate, base excess, IL-6, IL-18, hs-CRP, complications and outcome were compared based on the ISS. Patients were first compared as two groups based on the initial glucose levels (normoglycemic vs hyperglycemic). Then, all patients were divided into four subgroups based on the initial glucose and HbA1c values: (1) non-diabetic normoglycemic, (2) SIH, (3) diabetic normoglycemic and (4) DH (Fig. 1). The study groups were compared using χ^2^ test for categorical variables and the one-way analysis of variance (ANOVA) or Student’s t test for comparison of continuous variables whenever applicable. Yates’ corrected chi-square was used for categorical variables if the expected cell frequencies were below 5. A significant difference was considered when the two-tailed *p* value was less than 0.05. Freedmen ANOVAs, a nonparametric repeated measure analysis of variance, were performed separately for normoglycemia and hyperglycemia group to see the trend within the group for IL-6, IL-18, hs-CRP and blood glucose at baseline, after 24 h and 48 h, whereas Mann–Whitney U tests were used to see significant difference between the groups at each point of time and Wilcoxon signed-rank tests to see significant differences within each group from baseline for univariate exploratory analysis. Mixed model regression analyses were performed in the form of generalized estimating equations (GEE) to deal with longitudinal data and to adjust covariates. Cofactors as age and gender were adjusted along with groups (normoglycemia and hyperglycemia) and time variable for interleukin and hs-CRP values. Separate GEE analysis was performed using compound symmetry correlation structure.

Multivariable logistic regression analysis was performed to determine the odds ratio of on-admission blood glucose for the injury severity (ISS > 12) in one model after adjusting for age, shock index and blood transfusion and for hospital mortality in another model after adjusting for age, GCS, ISS, shock index and blood transfusion. Correlation of initial blood glucose level with respect to initial WBC count, lactate and base excess, ISS, RTS, TRISS, hospital length of stay and inflammatory markers was performed using Pearson’s correlation. Data analysis was carried out using the SPSS version 18 (SPSS Inc., Chicago, Illinois). Figures were plotted using GraphPad Prism software V.9.0 (La Jolla, CA).

## Results

### Patient and injury characteristics

During the study period, 250 trauma patients were enrolled. Figure 1 shows the study design. The vast majority of patients were males (98.0%) with mean age of 35.1 ± 10.1 years. The mean body mass index (BMI) was 26.3 ± 11.1 kg/m^2^, systolic blood pressure was 124.9 ± 20.7 mmHg and diastolic blood pressure was 78.2 ± 13.9 mmHg. The frequently injured body region was lower extremity (45.2%) followed by chest (40.4%), head (30.0%) and upper extremities (29.6%). The average glucose level at baseline was 8.38 mmol/l (95% CI; 8.01–8.74) and HbA1c was 5.61 (5.47–5.76). The mean ISS was 14.7 (95% CI 13.4–15.9), mean RTS score was 7.45 ± 1.13 and mean TRISS was 0.9566 ± 0.12. Blood transfusion was required in 28% cases and 3.2% developed in-hospital complications. The overall hospital mortality was 4.8% (12 patients).

### Normoglycemia *vs* hyperglycemia

In the trauma cohort (*n* = 250), 13% of the patients had hyperglycemia on their initial presentation, of which 50% had SIH and 50% had diabetic hyperglycemia.

Table 1 compares the demographics, clinical presentation and outcome of trauma patients according to the initial blood glucose levels (hyperglycemia versus normoglycemia). The two groups were comparable for age, gender and BMI. Compared with the normoglycemia group, hyperglycemic patients had higher HbA1c and positive hs-TnT (51.6% vs 16.2%; *p* = 0.001). In the hyperglycemia group, serum concentrations of IL-6 at baseline, at 24 h and 48 h post-trauma, were significantly higher than those of the normoglycemia group. Moreover, the hyperglycemia group had a significantly higher mean ISS 22.6 (95% CI 17.5–27.7 vs 13.5 (95% CI 12.3–14.7); *p* = 0.001), lower RTS (6.6 ± 1.7 vs 7.6 ± 0.9; *p* = 0.005) and TRISS scores (0.8681 ± 0.21 vs 0.9713 ± 0.10; *p* = 0.01) and required more blood transfusion (43.8% vs 25.7%; *p* = 0.03) than the normoglycemia group.

**Table 1:** Analysis of demographics, clinical presentation and outcome of trauma patients according to blood glucose level (October 2016–July 2019)

	Overall (*n* = 250)	Normoglycemia *n* = 218 (87%)	Hyperglycemia* *n* = 32 (13%)	p value
Age; years (*n* = 250) ^†^	35.1 ± 10.1	34.9 ± 10.1	36.7 ± 9.6	0.33
*Gender (n* = *250)*				
Males	245 (98.0%)	214 (98.2%)	31 (96.9%)	0.62
Females	5 (2.0%)	4 (1.8%)	1 (3.1%)	0.62
Body mass index (*n* = 248) ^†^	26.3 ± 11.1	25.8 ± 8.7	29.8 ± 20.9	0.29
hs-TnT positive (*n* = 235)	49 (20.9%)	33 (16.2%)	16 (51.6%)	0.001
Fasting glucose after 24 h (*n* = 247)**	7.25 (6.92–7.58)	6.9 (6.6–7.2)	9.2 (7.9–10.4)	0.001
Fasting glucose after 48 h (*n* = 237)**	6.38 (6.13–6.63)	6.2 (5.9–6.4)	7.5 (6.4–8.6)	0.006
Hemoglobin A1c % (*n* = 250)**	5.61 (5.47–5.76)	5.4 (5.3–5.5)	6.9 (6.1–7.8)	0.003
Injury Severity Score (*n* = 250)**	14.7(13.4–15.9)	13.5(12.314.7)	22.6(17.5–27.7)	0.001
Revised Trauma Score (*n* = 205) ^†^	7.45 ± 1.13	7.6 ± 0.9	6.6 ± 1.7	0.005
TRISS (*n* = 198) ^†^	0.9566 ± 0.1262	0.9713 ± 0.1011	0.8681 ± 0.2065	0.01
Hospital length of stay (*n* = 250) ^₤^	8 (2–146)	8 (2–68)	12.5 (3–146)**	0.001
ICU length of stay (*n* = 103) ^₤^	4 (1–40)	4 (1–40)	6 (2–23)	0.04
Blood transfusion (*n* = 250)	70 (28.0%)	56 (25.7%)	14 (43.8%)	0.03
Blood units transfused (*n* = 70) ^₤^	2.5 (1–68)	2 (1–22)	6 (1–68)	0.03
Hospital complications (*n* = 250)	8 (3.2%)	6 (2.8%)	2 (6.3%)	0.29
Hospital mortality (*n* = 250)	12 (4.8%)	8 (3.7%)	4 (12.5%)	0.02

^*^ Random blood glucose ≥ 11.1 mmol/l at baseline, TRISS: trauma injury severity score, Data presented as ^†^mean ± SD, or ** mean and 95% CI, ^₤^median and range. Statistical tests used: Student t tests and Mann–Whitney U tests were performed for interval variables between the two groups (Normoglycemia and Hyperglycemia) wherever applicable. Chi-square tests with Yate’s correction factors were used for categorical variables to see associations between the two groups

The hyperglycemia group had prolonged ICU (*p* = 0.001) and hospital stay (*p* = 0.001). Patients with hyperglycemia had higher mortality than normoglycemia group with 8.8% difference in death rate (12.5% *vs* 3.7%; *p* = 0.02).

Table 2 shows trend analysis of the laboratory parameters IL-6, IL-18 and hs-CRP, at three time points for each group. IL-6 values changes were significant between the normoglycemia and hyperglycemia groups and within the variable for each group. The IL-6 levels were significantly decreasing as time increasing for both the groups (*p* < 0.05), and the variable values were statistical more in the hyperglycemia than the normoglycemia group at each point of time (*p* < 0.05) (Table3 & Suppl Fig. 1). There was no statistically significant change between and within IL-18 levels. Notably, for hs-CRP levels, there were increases from the baseline to day 2 and 3 in the normoglycemia and hyperglycemia group (*p* < 0.05), however, there was no statistically significant difference at any point of time between the two groups.

**Table 2:** Trend analysis for Interleukin-6, Interleukin-18 and high-sensitive C-reactive protein (hs-CRP)

Variables	Overall	Normoglycemia	Hyperglycemia	*p* value between the twogroups
*Interleukin-6* (pg/ml)				
Baseline (on-admission) (*n* = 250)	137(121–153)	122(107–137)	240(172–308)	0.001
Second day (after 24 h) (*n* = 250)	120(101–138)	112(93–131)^a^	172(109–236)^g^	0.02
Third day (after 48 h) (*n* = 236)	108(90–125)	94(78–111)^b^	193(127–259)^k^	0.008
*p* value within the group	-	0.001	0.02	
*Interleukin-18* (pg/ml)				
Baseline (on-admission) (*n* = 250)	305(238–371)	299(226–372)	369(128–610)	0.11
Second day (after 24 h) (*n* = 250)	218(199–236)	218(197–238)^c^	217(186–248)^l^	0.28
Third day (after 48 h) (*n* = 236)	215(198–233)	211(192–231)^d^	242(194–291)^m^	0.05
*p* value within the group	–	0.64	0.20	
*Hs-CRP* (ng/ml)				
Baseline (on-admission) (*n* = 250)	3.86(3.45–4.26)	3.9(3.4–4.3)	3.9(2.7–5.1)	0.94
Second day (after 24 h) (*n* = 250)	9.50(9.03–9.97)	9.4(8.9–9.9)^e^	10.1(8.9–11.3)^n^	0.46
Third day (after 48 h) (*n* = 236)	9.64(9.14–10.13)	9.5(9.0–10.1)^f^	10.3(9.0–11.5)^o^	0.43
*p* value within the group	–	0.001	0.001	

^a^ Baseline vs Second day; *p* = 0.008, ^b^ Baseline vs third day; *p* = 0.001, ^c^ Baseline vs Second day; *p* = 0.17; ^d^ Baseline vs third day;*p* = 0.18, ^e^ Baseline vs Second day;*p* = 0.001, ^f^ Baseline vs third day; *p* = 0.001, ^g^ Baseline vs Second day;*p* = 0.009, ^k^ Baseline vs third day;*p* = 0.07, ^l^ Baseline vs Second day;*p* = 0.16, ^m^ Baseline vs third day; *p* = 0.51, ^n^ Second day; *p* = 0.001, ^o^ Baseline vs third day; *p* = 0.001. Baseline vs Data are given as mean and 95% confidence intervals within the group, whereas Mann–Whitney U tests were used to see significant difference between the groups at each point of time, and Wilcoxon signed-rank tests were used to see significance differences within each group from baseline for univariate exploratory within the group, whereas Mann–Whitney U tests were used to see significant difference between the groups at each point of time, and Wilcoxon signed-rank tests were used to see significance differences within each group from baseline for univariate exploratory analysis.

**Table 3:** GEE analysis for Interleukin-6 and hs-C-reactive protein (hs-CRP)

Parameter estimates							
Interleukin-6	*β*	Standard error	95% Wald confidence interval		Hypothesis test		
			Lower	Upper	Wald chi-square	df	*p *value
**1.** GEE analysis for interleukin-6 between and within factors							
(Intercept)	116.182	205.6032	− 286.793	519.157	0.319	1	0.572
[group = 0.00]	− 88.54	27.9884	− 143.395	− 33.683	10.007	1	0.002
[group = 1.00]	0^a^						
Gender	39.977	203.8760	− 359.613	439.566	0.038	1	0.845
Age	3.657	7.1711	− 10.398	17.712	0.260	1	0.610
(Scale)	18,002.161						

Parameter estimates							
Parameter	B	Std. error	95% Wald confidence interval		Hypothesis test		
			Lower	Upper	Wald chi-square	df	*p* value
2. GEE for hs-CRP between and within factors							
(Intercept)	12.977	4.3424	4.466	21.488	8.931	1	0.003
[group = .00]	− 0.477	0.4851	− 1.428	0.474	0.965	1	0.326
[group = 1.00]	0^a^						
Age	− 0.150	0.1039	− 0.354	0.053	2.088	1	0.148
Gender	− 4.860	4.3566	− 13.399	3.679	1.244	1	0.265
(Scale)	20.175						

Dependent Variable: Interleukin-6,

Model: (Intercept), group, Gender, Age, gender * age

a. Reference

Dependent Variable: Hs-C-reactive values

Model: (Intercept), group, Age, Gender, Age * Gender

a. Reference

Group 0 = normoglycemia, group 1 = hyperglycemia. Interleukin-6 and hs-CRP values were showing significant trend at univariate analysis. GEE multivariate mixed method was used to see trend analysis after adjusting age and gender confounders

### Analysis of the four subgroups

The four trauma patients’ groups were DH (*n* = 16), SIH (*n* = 16), diabetic normoglycemia (*n* = 11) and non-diabetic normoglycemia (*n* = 207) as shown in Table 4. Compared to the other three groups, patients with SIH were significantly younger (mean age 32 years), more obese, had severe injuries [mean ISS 24.5 (18–31)]; higher IL-6 levels at the three time points, prolonged hospital length of stay and higher mortality (*p* = 0.005). However, the four groups were comparable in terms of serum IL-18 and C-reactive protein levels at the different time points. Patients with diabetic hyperglycemia had higher hs-TnT and needed more units of blood transfusion. Higher shock index (> 0.80) was observed in both types of hyperglycemia compared to normoglycemic groups.

**Table 4:** Comparative analysis of trauma patients based on the initial blood glucose and hemoglobin A1C

	NN (*n* = 207)	ND (*n* = 11)	SIH (*n* = 16)	DH (*n* = 16)	*p* value
Age; years	34(33–36)	44(36–53)	32(28–36)	41(36–46)	0.001
Body mass index	26(24–27)	26(23–28)	34(19–50)	25(23–27)	0.030
Shock index	0.72(0.66–0.78)	0.71(0.55–0.87)	0.87(0.72–1.01)	0.88(0.69–1.07)	0.290
Injury Severity Score	13(12–14)	19(10.5–28)	24.5(18–31)	21(12–29)	0.001
Revised Trauma Score	8(7–8)	7(6–8)	6(5–7)	7(6–8)	0.001
GCS on arrival	14(13–14)	12(9–15)	10(7–13)	12(9–14)	0.001
HbA1c %	5.3(5.2–5.4)	7.3(6.02–8.65)	5.2(4.95–5.37)	8.7(7.52–9.81)	0.001
High-sensitive TroponinT	17(9–26)	12(9–15)	49(15–81)	114(-56–285)	0.003
*Interleukin-6* (pg/ml)					
On-admission	122(106–107)	130(55–204)	258(144–372)	222(135–308)	0.001
After 24 h	112(92–131)	123(51–195)	220(115–325)	125(49–201)	0.046
After 48 h	93(76–110)	113(20–207)	209(105–313)	176(83–269)	0.001
*Interleukin-18*(pg/ml)					
On-admission	301(229–373)	186(151–221)	200(153–246)	539(46–1031)	0.235
After 24 h	220(199–240)	233(151–315)	200(161–238)	235(184–287)	0.891
After 48 h	209(189–229)	244(150–338)	197(155–238)	288(200–376)	0.142
*Hs-CRP* (ng/ml)					
On-admission	3.9(3.4–4.3)	5.0(2.3–7.6)	4.3(2.1–6.5)	3.5(2.2–4.8)	0.642
After 24 h	9.6(9.1–10.1)	8.6(6.1–11.1)	10.6(9–12)	9.6(7.7–11.5)	0.601
After 48 h	9.6(9.0–10.1)	9(6–12)	10.9(9.3–12.4)	9.6(6.7-	0.576
TRISS	0.9733(0.96–0.99)	0.9359(0.85–1.01)	0.8207(0.67–0.97)	0.9092(0.82–0.99)	0.001
Blood transfusion	26.1%	18.2%	56.3%	31.3%	0.062
Blood units transfused	3(3–4)	13(-101–127)	10(1–19)	18(-17–53)	0.002
Any surgical intervention	58%	45%	44%	44%	0.430
Hospital length of stay	12(10–14)	10(5–15)	26(7–44)	18(9–27)	0.003
ICU length of stay	6(4–7)	10(2–17)	10(6–14)	8(2–14)	0.106
Any hospital complications (*n* = 8)	2.9%	0%	6.3%	6.3%	0.709
Hospital mortality (*n* = 12)	2.9%	18.2%	18.8%	6.3%	0.005

*NN*, normoglycemia non-DM: *ND*, normoglycemia DM: *SIH*, stress-induced hyperglycemia: *DH*, diabetic hyperglycemia: Continuous variables are expressed as mean and 95% confidence intervals. Comparative analysis of trauma patients based on the initial blood glucose and hemoglobin A1C was performed using one-way ANOVA tests for interval variables and chi-square tests for categorical variables

**Fig. 2:**
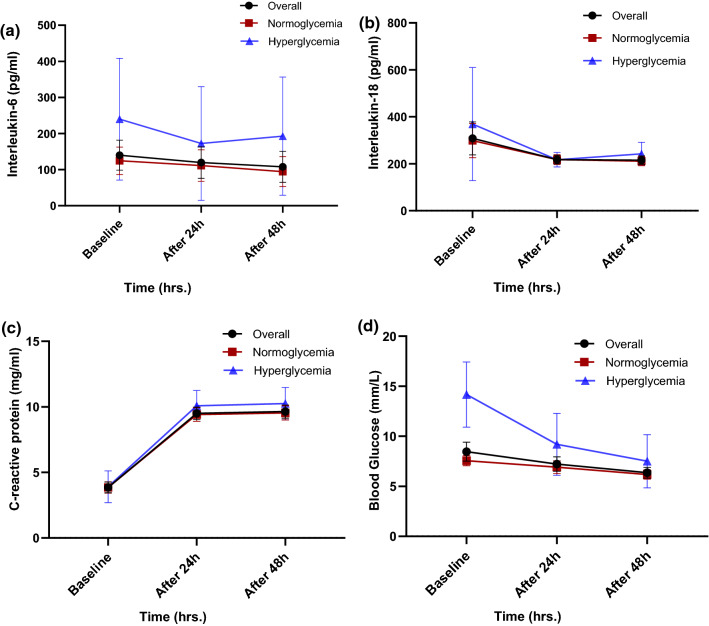
Trend of inflammatory markers and blood glucose levels in the overall, normoglycemic and hyperglycemic trauma patients: **a** IL-6; **b** IL-18; **c** C-reactive protein; and **d** blood glucose levels

Figure 2 demonstrates the trend of inflammatory markers and blood glucose levels in trauma patients. The serum levels of IL-6, IL-18 and blood glucose increased after injury then showed a slowly decreasing trend, but did not reach baseline after 48 h.

Table 5 illustrates the bivariate correlation between blood glucose levels and other factors. Significant positive correlations were identified between initial blood glucose level and serum lactate (*r* = 0.467, *p* = 0.001), ISS (*r* = 0.368, *p* = 0.001), IL-6 (*r* = 0.373, *p* = 0.001) and hospital length of stay (r = 0.304, *p* = 0.001), whereas blood glucose levels showed significant negative correlations with base excess (*r* = −0.417, *p* = 0.001), GCS score (*r* = −0.306, *p* = 0.001), TRISS (*r* = −0.310, *p* = 0.001) and RTS (*r* = −0.354, *p* = 0.001). IL-6 correlated well with ISS (*r* = 0.40, *p* = 0.001).

**Table 5:** Bivariate correlation between blood glucose and other factors

Parameters	Pearson’s correlation	*p* value
Random glucose vs WBC at ED	0.244	0.001
Random glucose vs Lactate at ED	0.467	0.001
Random glucose vs Base Excess at ED	− 0.417	0.001
Random glucose vs GCS at ED	− 0.306	0.001
Random glucose vs ISS	0.368	0.001
Random glucose vs TRISS	− 0.310	0.001
Random glucose vs RTS	− 0.354	0.001
Random glucose vs Hospital LOS	0.304	0.001
Random glucose vs Interleukin-6*	0.373	0.001
Random glucose vs Interleukin-18*	0.108	0.09
Random glucose vs hs-CRP*	− 0.059	0.35

^*^ Baseline; *RTS* Revised trauma score, *TRISS* Trauma injury severity score, *ISS* Injury severity score, *hs-CRP* High-sensitive C-reactive protein, *ED* Emergency department**,** *LOS* Length of stay, Bivariate correlation coefficients were calculated between glucose and other factors

Table 6 shows the laboratory parameters and outcomes based on ISS. Compared to mild and moderate injury, patients sustained severe injury (ISS ≥ 16) were more likely to have higher level of blood glucose, serum lactate, base excess and IL-6 (*p* = 0.001 for all). However, the mean serum levels of IL-18 and hs-CRP, at three time points, did not significantly differ among the ISS groups.

**Table 6:** Presentation of inflammatory markers and glucose level based on Injury Severity Score (ISS)

Parameters	Mild (ISS ≤ 8) *n* = 53	Moderate (ISS 9–15) *n* = 112	Severe (ISS ≥ 16) *n* = 84	*p* value
*Blood glucose level* (mmol/l)				
Random (on admission)	7.21 ± 1.66	8.05 ± 2.69	9.59 ± 3.45	0.001
Fasting (after 24 h)	6.65 ± 1.41	6.99 ± 1.75	7.85 ± 3.05	0.004
Fasting (after 48 h)	6.11 ± 1.30	6.36 ± 1.46	6.49 ± 2.17	0.48
HbA1c %	5.35 ± 0.55	5.61 ± 1.14	5.76 ± 1.44	0.14
*Serum lactate*; mmol/L				
Baseline (on-admission)	2.71 ± 1.34	2.71 ± 1.11	3.29 ± 1.77	0.01
Second day (after 24 h)	1.82 ± 0.95	1.64 ± 0.85	2.18 ± 1.86	0.02
Third day (after 48 h)	1.22 ± 0.72	1.25 ± 0.52	1.48 ± 1.45	0.24
*Base Excess*				
Baseline (on-admission)	− 2.01 ± 3.06	− 1.85 ± 2.45	− 4.67 ± 4.19	0.001
Second day (after 24 h)	− 0.75 ± 2.00	− 0.72 ± 2.46	− 3.03 ± 3.84	0.001
Third day (after 48 h)	1.40 ± 2.34	1.01 ± 2.53	− 1.79 ± 4.50	0.001
*Interleukin-6* (pg/ml)				
Baseline (on-admission)	61.4 (5.8–510.3)	75.9 (4.8–595.9)	146.6 (4.02–627.1)	0.001
Second day (after 24 h)	47.6 (3.1–374.1)	48.6 (6.7–722.7)	85.6 (5.3–1109.3)	0.001
Third day (after 48 h)	37.3 (2.4–547.9)	48.6 (1.9–550.0)	74.5 (3.2–550.0)	0.04
*Interleukin-18* (pg/ml)				
Baseline (on-admission)	191.6 (51.9–1214.3)	186.8 (32.4–3890.9)	197.5 (45.0–5000.0)	0.94
Second day (after 24 h)	184.9 (50.6–1328.9)	183.5 (41.3–739.9)	211.1 (47.1–737.4)	0.20
Third day (after 48 h)	170.3 (30.3–785.6)	178.5 (1.04–947.9)	197.7 (55.0–705.1)	0.07
*hs-CRP* (ng/ml)				
Baseline (on-admission)	3.05 (0.06–13.1)	3.09 (0.18–13.5)	3.35 (0.03–12.8)	0.55
Second day (after 24 h)	10.8 (0.8–14.9)	10.8 (0.007–14.9)	10.6 (0.09–13.9)	0.22
Third day (after 48 h)	10.9 (0.02–15.5)	10.9 (0.004–14.3)	10.7 (0.2–14.9)	0.56
All complications	1 (1.9%)	0 (0.0%)	7 (8.3%)	0.004
Hospital length of stay; days	6.0 (2–62)	6.5 (2–68)	11.5 (2–146)	0.001
In-hospital mortality	0 (0.0%)	0 (0.0%)	12 (14.3%)	0.001

One-way ANOVA for interval variables and Chi-square test

Multivariable logistic regression analysis showed that on-admission hyperglycemia had an adjusted odds ratio (aOR) 2.42 (95% CI 1.076–5.447, *p* = 0.03) for severe injury after adjusting for age, shock index and blood transfusion. However, it was not predictor for hospital mortality (aOR 0.93; 95% CI 0.143–6.102, *p* = 0.94) after adjusting for age, shock index, ISS, GCS and transfusion (Table 7).

**Table 7:** Multivariable logistic regression analysis for predictors of injury severity and in-hospital mortality

Variable	*p* value	Odds ratio	95% confidence interval
a-Predictors of injury severity (ISS > 12)			
Age; years	0.642	1.006	0.981–1.032
Shock index	0.523	0.786	0.376–1.647
Blood transfusion	0.126	1.562	0.882–2.765
Admission hyperglycemia	0.033	2.421	1.076–5.447
b-Predictors of hospital mortality			
Age; years	0.041	1.088	1.003–1.179
Blood transfusion	0.695	0.680	0.099–4.683
Admission hyperglycemia	0.943	0.934	0.143–6.102
Glasgow Coma Scale on admission	0.001	0.731	0.615–0.869
Injury Severity Score	0.008	1.100	1.025–1.180
Shock index	0.092	2.135	0.883–5.167

## Discussion

This is a prospective study to identify the patterns and effect of initial hyperglycemia and inflammatory biomarkers in trauma patients. Up to our knowledge, the constellation of on-admission random blood glucose, proinflammatory cytokines and injury severity scale among trauma patients is understudied. There are several key findings in this study. Almost 13% of trauma patients had on-admission hyperglycemia; half of them had SIH. Patients presented with hyperglycemia were more likely to have higher levels of IL-6, IL-18 and hs-CRP compared to the normoglycemic patients. Also, patients with hyperglycemia were more likely to have severe injuries, prolonged hospitalization and higher mortality than normoglycemic patients. Moreover, IL-6 levels were greater in SIH (non-diabetic) compared to DH group. Logistic regression analysis showed that adjusted on-admission blood glucose was a predictor for injury severity and not for hospital mortality.

Furthermore, this study showed that the initial readings of IL-6 correlated significantly with the patient ISS. The blood glucose levels showed significant correlation with serum IL-6, serum lactate, ISS and length of hospitalization. Also, we observed an association between initial hyperglycemia and hs-TnT. Prior works revealed a significant association between the severity of trauma and positivity of hs-TnT as a reflection of traumatic stress [21, 22]. Also, patients with initial hyperglycemia had a higher shock index on admission (> 0.80). Prior studies reported that trauma patients with high SI value had worse presentation and outcomes [23, 24]. Of note, the number of transfused blood units was higher in DH and SIH than normoglycemic groups in our study. Kreutziger et al., in their retrospective study, found that the rate of hemorrhagic shock was increasing with the increase in the on-admission blood glucose levels [4]. Although our study and Kreutziger et al. study had almost a similar sample size, our study was prospective study, looked for four different glycemic conditions and explored the correlation between hyperglycemia, cytokines, CRP and ISS.

The current study demonstrated that patients with SIH had a threefold higher rate of mortality as compared to those with DH. These findings are in accordance with earlier studies which showed significantly greater risk of mortality in patients with SIH as opposed to those who had DH [11, 15, 16, 25].

A recent study on thoracoabdominal injury patients demonstrated a higher rate of mortality in non-diabetic patients with on-admission hyperglycemia as compared to those with initial normoglycemia [26]. Furthermore, a prospective observational study of traumatic brain injury (TBI) reported a marked hyperglycemia in patients with severe TBI which independently predicted the poor short-term neurological outcome [27].

Prior studies showed a relationship between serum cortisol, catecholamine levels and severity of injury. Patients with severe injuries were more likely to develop SIH [5, 28–30]. Consistent with these observations, hyperglycemia patients in our cohort had a higher injury severity as compared to normoglycemic patients.

In our study, the overall complications were higher in both types of hyperglycemia compared to normoglycemia groups, but the difference did not reach statistical significance. A prior study reported that initial hyperglycemia in trauma patients correlated with serum lactate and ISS and was associated with higher mortality; however, the rate of infection was not significantly higher [31]. The mortality in the present study cohort is 4.8% which is consistent with the overall in-hospital mortality among trauma patients in Qatar (4.3%) between 2010 and 2018. [32]

In our patients, the increase in hs-CRP was detected after 24 h of trauma and reached its peak value at 48 h. Giannoudis et al. [33] reported that serum CRP levels were within the normal range on the initial presentation which then gradually increased and reached the peak value on the third day post-trauma. The authors also found an association between ISS and IL-6 levels but such association was not observed with CRP. Consistent with our study, earlier studies reported a significant correlation between higher ISS and IL-6 level on the initial presentation [34–36]. We also observed a higher level of IL-18 in DH in comparison with the other groups including SIH (but statistically non-significant); a finding that needs further explanation [37]. Our study performed serial measurements of blood glucose and cytokines to understand the complex relationship between hyperglycemia and inflammatory response in trauma. These findings indicated that immunoneuroendocrine alterations might be involved in the pathophysiology of trauma patients [14, 15, 20].

### Limitations

The first limitation is that patients with DH may have also some degree of stress response which was underestimated. Second, we could not measure the levels of stress response hormones or catecholamines. Third, selection bias cannot be ignored along the study period. The impact of hyperglycemia in female patients was not well studied as 98% of the study subjects were males. This figure reflects the gender discrepancy in trauma patients in Qatar as 90% of all trauma admissions in previous reports were males [38, 39]. Furthermore, based on the Qatar’s mid-year population estimates (2014–2017), females make up almost a quarter of the total country population [40]. In the emergency department, we were using sliding scale insulin for patients with hyperglycemia; however, we did not measure the effect of exogenous insulin on the cytokine’s levels. Apart from the significant difference in age, we could not explain the mortality rate in the normoglycemic diabetic group which was relatively similar to that of the SIH. Finally, the HbA1c level might not be accurate in patients who necessitate early blood transfusion as transfusion may alter the HbA1c reading [41, 42]. Of note, HbA1c value was reported as % according to the Diabetes Control and Complications Trial units as we are not using the mmols/mol values (International Federation of Clinical Chemistry units) in our laboratory.

## Conclusion

Patients with on-admission hyperglycemia have more severe injury and worse hospital outcome compared to normoglycemia patients. The initial blood glucose correlates with serum IL-6 which indicates a potential role of the systemic inflammatory response in the disease pathogenesis among severely injured patients. On-admission glucose level could be a useful marker of injury severity, triage and risk assessment in trauma patients. These observations warrant further evaluation in larger multicenter studies.

## Supplementary Information

Below is the link to the electronic supplementary material.

Supplementary file1 (DOC 149 KB)

## Data Availability

All data were shown in the study analysis and tables.

## Abbreviations

ISS: Injury severity score
hs-CRP: High-sensitive reactive protein
IL: Interleukin
HbA1c: Glycosylated hemoglobin
SIH: Stress-induced hyperglycemia
DH: Diabetic hyperglycemia
