# Supplementary material for: Patterns and Effects of Admission Hyperglycemia and Inflammatory Response in Trauma Patients: A Prospective Clinical Study
Source: World J Surg. 2021 Jun 11;45(9):2670–81. doi: 10.1007/s00268-021-06190-5 (PMC8321976; doi:10.1007/s00268-021-06190-5)

Suppl Figure1 : correlation structure with IL-6 at 3 time points (A with B and C).


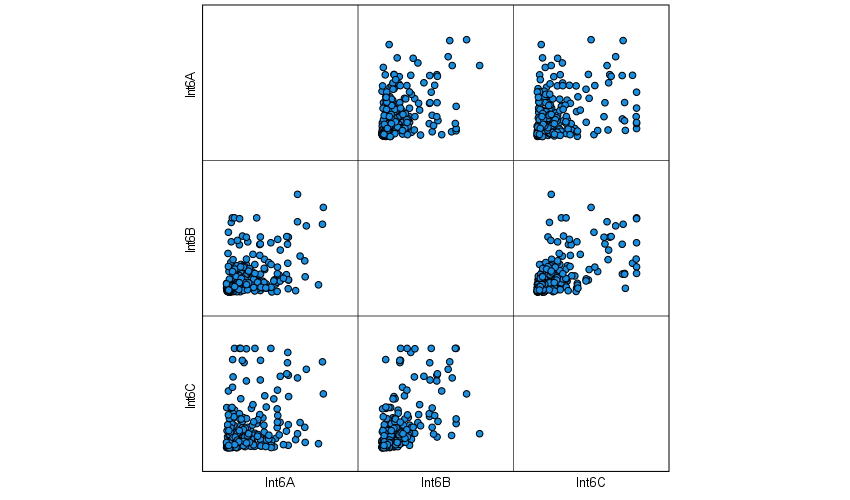


| Correlation structure for Hs-C-reactive protein (ng/ml) parameter. |
| --- |


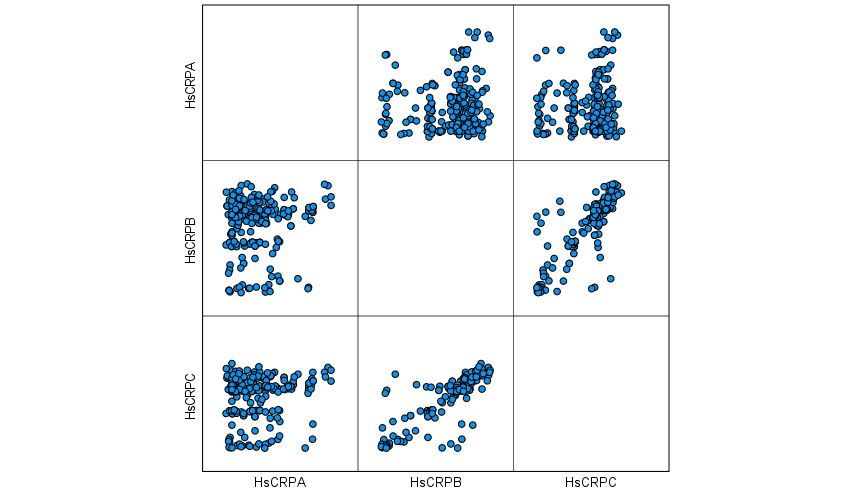

Supplement: Supplementary file 1 — Supplementary file1 (DOC 149 KB) [file 268_2021_6190_MOESM1_ESM.doc]
